# Atractylodin Attenuates Dextran Sulfate Sodium-Induced Colitis by Alleviating Gut Microbiota Dysbiosis and Inhibiting Inflammatory Response Through the MAPK Pathway

**DOI:** 10.3389/fphar.2021.665376

**Published:** 2021-07-15

**Authors:** Linghang Qu, Xiong Lin, Chunlian Liu, Chang Ke, Zhongshi Zhou, Kang Xu, Guosheng Cao, Yanju Liu

**Affiliations:** ^1^ College of Pharmacy, Hubei University of Chinese Medicine, Wuhan, China; ^2^ Center for Hubei TCM Processing Technology Engineering, Wuhan, China

**Keywords:** ulcerative colitis, malonylation, atractylodin, MAPK pathway, tight junctions

## Abstract

In this study, we investigated the therapeutic effects and mechanism of atractylodin (ATL) on dextran sulfate sodium (DSS)-induced ulcerative colitis in mice. We found that atractylodin could significantly reverse the effects of DSS-induced ulcerative colitis, such as weight loss, disease activity index score; shorten the colon length, and reverse the pathological changes in the colon of mice. Atractylodin could inhibit the activation of colonic macrophages by inhibiting the MAPK pathway and alleviate intestinal inflammation in the mouse model of ulcerative colitis. Moreover, it could protect the intestinal barrier by inhibiting the decrease of the tight junction proteins, ZO-1, occludin, and MUC2. Additionally, atractylodin could decrease the abundance of harmful bacteria and increase that of beneficial bacteria in the intestinal tract of mice, effectively improving the intestinal microecology. In an LPS-induced macrophage model, atractylodin could inhibit the MAPK pathway and expression of the inflammatory factors of macrophages. Atractylodin could also inhibit the production of lactate, which is the end product of glycolysis; inhibit the activity of GAPDH, which is an important rate-limiting enzyme in glycolysis; inhibit the malonylation of GAPDH, and, thus, inhibit the translation of TNF-α. Therefore, ours is the first study to highlight the potential of atractylodin in the treatment of ulcerative colitis and reveal its possible mechanism.

## Introduction

Ulcerative colitis (UC) is a chronic inflammatory bowel disease of unknown etiology that affects the colon and rectum. It is one of the two forms of inflammatory bowel disease (IBD). The characteristic of UC is that mucositis begins at the rectum and extends progressively to the proximal colon (Kobayashi et al., 2020). Of late, the global incidence of UC is steadily increasing. The incidence of UC in western Canada is the highest (16.7 per 100,000 individuals), while in Europe, it is 1.6–11.9 per 100,000 individuals (Kaur and Goggolidou, 2020). Currently, the pharmacotherapy of UC is based mainly on the following four basic drug categories: 5-aminosalicylates (5-ASA), steroids, immunosuppressants, and biological drugs (Panés et al., 2017). Local mesalazine continues to be the first-line treatment for patients with mild or moderate active UC limited to the rectum, whereas oral and local 5-ASA are the main treatment approaches for patients with left or extensive colitis (Cohen and Dalal, 2015). A study reports that only 60% of patients with mild to moderate UC achieved remission with mesalazine (Ford et al., 2011). Anti-TNF drugs may increase the risk of severe infection and cancer (Fellermann, 2013). Therefore, there is an urgent need for the effective management of ulcerative colitis; consequently, an increasing number of studies aimed at discovering novel and effective natural products are being conducted.

In patients with UC, several pro-inflammatory factors, such as tumor necrosis factor (TNF), interleukin-6, and interleukin-1*β* are produced in the intestinal mucosa (Tatiya-aphiradee et al., 2018). Intestinal goblet cells secrete MUC2 may be the key factor in determining the susceptibility of an individual to UC (Morampudi et al., 2016). The mucous layer of the colon in patients with UC is characterized by a decreased synthesis of mucoprotein 2 (Van Klinken et al., 1999). The barrier function of the intestinal epithelium is partly maintained by tight junctions (TJs) present between adjacent epithelial cells. TJs constitute a complex functional compound that is mainly composed of TJ proteins. Abnormal expression of TJ proteins (ZO-1 and occludin) in intestinal tissues promotes intestinal permeability and pathogen infiltration, and induces immune dysfunction and IBD (Zeisel et al., 2018). Macrophages are the primary immune cells that play a key role in the development, progression, and reversal of inflammation (Fujiwara and Kobayashi, 2005). There is a causal relationship between the regression of intestinal inflammation and the differentiation of monocyte macrophages in patients with IBD. Macrophages are now considered a potential target in the development of novel therapeutic methods (Na et al., 2019). The activation of macrophages is closely related to the MAPK pathway (Smith et al., 2014). The expression of phosphorylated p38 MAPK in the nucleus of immune effector cells in the UC mucosal crypt is significantly increased (Dahan et al., 2008). There is evidence that the intestinal microflora of patients with IBD is significantly different from that of healthy individuals. Although clinical data are limited, they support the efficacy of treatment strategies for altering the microbiota of patients with IBD (Plichta et al., 2019).

Several natural products isolated from vegetables, fruits, and herbs have been reported to be effective in treating dextran sulfate sodium (DSS)-induced ulcerative colitis in mice. For example, 2-O-6-D-glucosyl-L-ascorbic acid from *Lycium barbarum* can increase the expression of the intestinal tight junction proteins, ZO-1, and occludin, and regulate the diversity of intestinal short-chain fatty acids and intestinal flora, thereby being effective in the treatment of DSS-induced colitis (Dong et al., 2020). Parthenolide can regulate the balance of intestinal flora and suitably control the levels of short-chain fatty acids and Treg/Th17 in the intestinal mucosa to prevent UC (Liu et al., 2020). Paeoniflorin can reduce the infiltration of Gram-positive bacteria in the intestinal tract and inhibit the MDP-NOD2 pathway, which is dependent on Gram-positive bacteria, effectively reducing colitis in mice (Luo et al., 2021). Physalin B significantly improves the clinical symptoms and signs of DSS-induced UC mice, and reduces the loss of body weight as well as the shortening of colon length (Zhang et al., 2020).

Among these compounds, we were more interested in atractylodin, a natural compound obtained from Atractylodes, which is known for its anti-inflammatory effects. Intraperitoneal injection of atractylodin can significantly reduce the severity of disease progression in mice with rheumatoid arthritis, which is manifested by the reduction of paw swelling, clinical arthritis score, and histopathological changes in the joint (Chuang et al., 2019). Atractylodin not only significantly reduces the histopathological changes during lipopolysaccharide (LPS)-induced acute lung injury but also decreases myeloperoxidase activity, wet/dry weight ratio of the lung, protein leakage, and inflammatory cell infiltration (Tang et al., 2018). However, whether atractylodin can improve DSS-induced ulcerative colitis in mice and whether it can regulate the abundance of intestinal microorganisms have not been reported. It is also unclear whether the anti-inflammatory effect of atractylodin is related to the inhibition of macrophage activation. Therefore, we performed *in vivo* and *in vitro* studies by using a DSS-induced mouse model of UC and LPS-stimulated RAW264.7 cells, respectively, investigated the effects of atractylodin on macrophage activation, and evaluated if it could be a potential target for UC treatment.

## Materials and Methods

### Reagents and Chemicals

Atractylodin (purity > 98%) was purchased from Chengdu Push Biotechnology Co., Ltd (Chengdu, China). Sulfasalazine was procured from Shanghai Sine Tianping Pharmaceutical Co., Ltd (Shanghai, China). DSS (36,000–50,000 Da) was obtained from MP Biomedicals (Solon, OH, United States). Dimethyl sulfoxide and LPS O55:B5 were purchased from Merck (Darmstadt, Germany). Mouse TNF-α ELISA kit was purchased from Elabscience Biotechnology Co., Ltd. (Wuhan, China). Lactate assay kit was obtained from Nanjing Jiancheng Bioengineering Institute. GAPDH assay kit was purchased from GENMED Biotechnology Co., Ltd. (Shanghai, China). Pierce co-immunoprecipitation kit was purchased from Thermo Scientific (Waltham, MA, United States). Antibodies against P38 (#9212S), P-P38 (#4511S), P44/42 MAPK (ERK 1/2) (#4695S), P-P44/42 MAPK (T202/Y204) (#9101S), SAPK/JNK (#9252S), and P-SAPK/JNK (T183/Y185) (#4668S) were purchased from CST (Boston, MA, United States). Antibodies against GAPDH (#60004-1-Ig), IL-1β (#16806-1-AP), and IL-6 (#66146-1-Ig) were purchased from Proteintech (Wuhan, China). Antibodies against *β*-actin (#K200058^M^) was purchased from Solarbio (Beijing, China). MUC2 antibodies (#ab97386) were purchased from Abcam Inc. (Cambridge, MA, United States). Antibodies against TNF-α (#AF7014) were purchased from Affinity (Liyang, China). Anti-Kmal antibody (#PTM-901) was purchased from PTM Biotechnology Co., Ltd. (Hangzhou, China).

### Animal Experiments

Specific-pathogen-free male BALB/c mice (6–8-weeks old, 18–22 g) were purchased from the Experimental Animal Center of China Three Gorges University (Yichang, China) (animal license No. SYXK (E) 2017–0,067). BALB/c mice were housed in SPF animal room with temperature of 24 ± 1°C and humidity of 50–70%. They were subjected to a 12 h:12 h light:dark cycle and allowed to acclimatize to their surroundings for one week. Forty mice were randomized into the following five groups: normal, model, atractylodin-10 mg/kg, atractylodin-20 mg/kg, and Sulfasalazine (SASP), with eight mice per group. From the first to the eighth day, the mice in the normal group were given free access to pure drinking water, whereas those in the other groups were provided access to a 3.5% DSS solution. The pure water and DSS solution were changed every two days. From the first to the seventh day, the mice in the atractylodin group were intraperitoneally injected atractylodin at a dose of 10 or 20 mg/kg, whereas those in the SASP group were intragastrically administered 250 mg/kg of sulfasalazine. On the eighth day, all mice were anesthetized using pentobarbital sodium and blood were withdrawn. The colons of mice were photographed and their lengths were measured. A 1 cm long piece of the colon was cut from 1 cm below the cecum for staining, immunohistochemistry, and immunofluorescence. The rest of the colon was reserved for western blotting.

### Histological Evaluation

Mice were sacrificed and the colon sections were rinsed with ice-cold phosphate-buffered saline (PBS). The excess PBS was blotted and the tissue samples were immediately fixed in 10% buffered formalin, dehydrated, and embedded in paraffin. Sections (5 μm thick) were stained using hematoxylin and eosin.

### Alcian Blue-Periodic Acid Schiff

Paraffin sections of mice colon were treated with xylene I for 20 min, xylene II for 20 min, absolute ethanol I for 5 min, absolute ethanol II for 5 min, and 75% alcohol for 5 min, followed by washing with tap water. The sections were stained with AB-PAS C for 15 min and rinsed with tap water until colorless. Next, the sections were stained with AB-PAS B for 15 min, rinsed once with tap water, and twice with distilled water. AB-PAS A was allowed to equilibrate to room temperature, after which the sections were stained for 30 min in the dark and then rinsed for 5 min. The sections were further treated with anhydrous ethanol I for 5 min, anhydrous ethanol II for 5 min, anhydrous ethanol III for 5 min, xylene I for 5 min, and xylene II for 5 min. The transparent slices were sealed with neutral gum and observed using microscopy for image acquisition and analysis.

### Disease Activity Index

Individual scores were combined to generate the DAI, which was calculated daily for each mouse. The maximum score was 12 based on a 0–4 scoring system for the following parameters: Score = 0, Weight loss: none, Stool consistency: normal, Blood in stool: none; Score = 1, Weight loss: 1–5%, Stool consistency: loose stools, Blood in stools: presence of blood; Score = 2, Weight loss: 5–10%, Stool consistency: watery diarrhea, Blood in stools: presence of blood; Score = 3, Weight loss: 10–20%, Stool consistency: slimy diarrhea, little blood, Blood in stools: presence of blood; Score = 4, Weight loss: > 20%, Stool consistency: severe watery diarrhea with blood, Blood in stools: gross bleeding (Liu et al., 2020).

### Immunohistochemistry

Colon tissue was fixed in 4% (w/v) paraformaldehyde solution, embedded in paraffin, sectioned, dewaxed, and then treated with the antigen. Next, the samples were incubated with primary and secondary antibodies at 4°C. Subsequently, the nuclei were stained using DAPI (Beyotime, Nanjing, China), and the sections were imaged using an Olympus FV 1000 laser confocal microscope (Olympus, Tokyo, Japan) (Shi et al., 2019).

### Immunofluorescence

Immunostaining was performed according to previously published protocols (Liu et al., 2020). The primary antibodies F4/80 (Affinity, 28463-1-AP), iNOS (Affinity, 18985-1-AP), ZO-1 (Abcam, ab216880), and occludin (Abcam, ab167161) were used. After incubation with the corresponding secondary antibodies, the cell nucleus was stained using DAPI (Roche, Shanghai, China) for 15°min, following which, the samples were observed and the images were captured using fluorescence microscopy (Olympus, Tokyo, Japan).

### Cell Culture and Drug Administration

RAW264.7 cells were purchased from the Cell Bank of the Chinese Academy of Sciences (Shanghai, China). Cells were cultured in DMEM with 10% fetal bovine serum, 1% nonessential amino acids, and 1% penicillin-streptomycin and incubated in an environment of 5% CO_2_ at 37°C. RAW264.7 cells were seeded in 6-well plates or Petri dishes and divided into normal, model, low-dose atractylodin, medium-dose atractylodin, and high-dose atractylodin groups. The low, medium, and high-dose groups were stimulated with LPS for 24 h and treated with 10, 20, and 40 μM of atractylodin, respectively. The normal group was not stimulated with LPS, whereas the model group was subjected to LPS stimulation; both the normal and model groups were not subjected to atractylodin intervention.

### RT-PCR

Trizol reagent was used to collect cells from the 6-well plates (Guangzhou Jet Bio-Filtration Co., Ltd: TCP-010–006), after which RNA was extracted and purified. The cDNA of each sample was reverse transcribed using a commercial kit (Vazyme Biotech Co., Ltd., Nanjing, China). Then, the reverse transcription product was used as a template to perform real-time polymerase chain reaction (PCR) using a Step One Plus thermal cycler and PowerUp™ SYBR™ Green Master Mix (Vazyme Biotech) following the manufacturer’s instructions. All the primers were referenced from the previous study and synthesized by Invitrogen. The primer sequences are shown in Supplementary Table S1. The final data were analyzed using the 2^-∆∆CT^ method.

### Western Blotting

Western blotting was performed according to standard protocols. Briefly, mice colon tissues were homogenized and lyzed with a cleavage buffer containing the cocktail. The total protein was separated using SDS-PAGE and transferred to a polyvinylidene fluoride membrane. The membrane was sealed with skim milk for 2°h, incubated with the primary antibody overnight, and then incubated with the secondary antibody at room temperature for 2 h. An ECL chemiluminescence detection kit was used to detect the protein bands.

### Enzyme-Linked Immunosorbent Assay

The cell-culture medium was collected and centrifuged at 1,000 x*g* for 20 min at 4°C. The supernatant was used to detect the concentration of TNF-α according to the manufacturer’s instructions provided in the ELISA kit (Linghang et al., 2020).

### Co-Immunoprecipitation

The cell protein lysates were collected, 3 μM trichostatin A (GLPBIO, Montclair, United States) and 50 mM nicotinamide (Merck, Darmstadt, Germany) were added for protein extraction. First, the GAPDH antibody was immobilized on the coupling resin and then the control agarose resin was used to pretreat the cell lysate. Next, the GAPDH-immobilized antibody was used for immunoprecipitation. After elution, the malonylated antibody was used for SDS-PAGE detection.

### Determination of Lactate

The supernatants of RAW264.7 cells treated for 24 h and untreated cells were collected and centrifuged at 1,000 x*g* for 20 min at 4°C to determine the lactate levels according to the manufacturer’s instructions provided in the lactate assay kit (Nanjing Bioengineering Institute, Nanjing, China). First, 20 μL of sample, 1 ml of working enzyme solution, and 200 μL of chromogenic agent were added to a test tube. Then, the mixture was vortex mixed and incubated at 37°C for 10 min. Then, 2 ml of termination reagent was added to arrest the reaction. Lastly, the OD value was determined at 530 nm using a spectrophotometer.

### Determination of GAPDH Activity

GAPDH activity was determined using colorimetry. Briefly, 3 ml of GENMED cleaning solution was added to both treated and untreated RAW264.7 cell cultures to rinse the cell surface after 24 h of treatment. Next, 3 ml of GENMED cleaning solution was added to the cells and centrifuged at 300 x*g* for 5 min at 4°C. The supernatant was discarded and 500 μL of GENMED lysis solution was added, mixed well, vortexed for 15 s, incubated in ice for 30 min, and centrifuged at 16,000 x*g* for 5 min at 4°C. Next, 500 μL of the supernatant was taken to 1.5 ml tube. About 10 μL of the sample was used for protein quantification. Briefly, 150 μL of GENMED buffer solution was taken in a 96-well plate and 20 μL of GENMED reaction solution was added to each well. Then, 20 μL of GENMED substrate solution was added, mixed well, and incubated at 25°C for 3 min. Lastly, GENMED negative solution or a sample containing 20 μg of protein was added to the corresponding wells and mixed thoroughly. Absorbance of the samples was determined at 340 nm using a microplate reader.

### 16SRNA Sequencing

Fecal genomic DNA was extracted from 0.1 g of frozen fecal samples using cetyltrimethyl ammonium bromide or sodium dodecyl sulfate and the purity and concentration were determined using agarose gel electrophoresis. Amplicon libraries covering the V3–V4 hypervariable regions of the bacterial 16S-rDNA gene were amplified using primers 338F: 5′-ACT​CCT​ACG​GGA​GGC​AGC​A-3′, and 806R: 5′-GGACTACHVGGGTWTCTAAT-3′. PCR was performed using a 25 μL mixture containing 5 μL of 5× buffer, 2 μL of 2.5 mM dNTPs, 1 μL of each primer (5 μM), 0.25 μL of Fast Pfu polymerase, 1 μL of template DNA, 14.75 μL of ddH_2_O. PCR was conducted as follows: initial denaturation for 3 min at 95°C followed by 27 cycles of 30 s at 95°C, 30 s for annealing at 55°C, 45 s for elongation at 72°C, and a final extension at 72°C for 10 min. PCR products were detected using 2% agarose gel electrophoresis. The library was constructed using TruSeq Preparation DNA PCR-Free sample preparation kit. The library was quantified using Qubit and Q-PCR, qualified, and sequenced using NovaSeq 6,000.

### Statistical Analysis

Statistical analysis was performed using GraphPad v8.0. Data are presented as mean ± standard deviation. Student’s *t*-test or one-way ANOVA followed by Bonferroni test was used to compare two independent variables; ns, not significant, ^#^ *p* < 0.05, ^##^ *p* < 0.01, *p < 0.05, **p < 0.01.

## Results

### Atractylodin can Improve the Pathological Symptoms of UC in Mice

**FIGURE 1:**
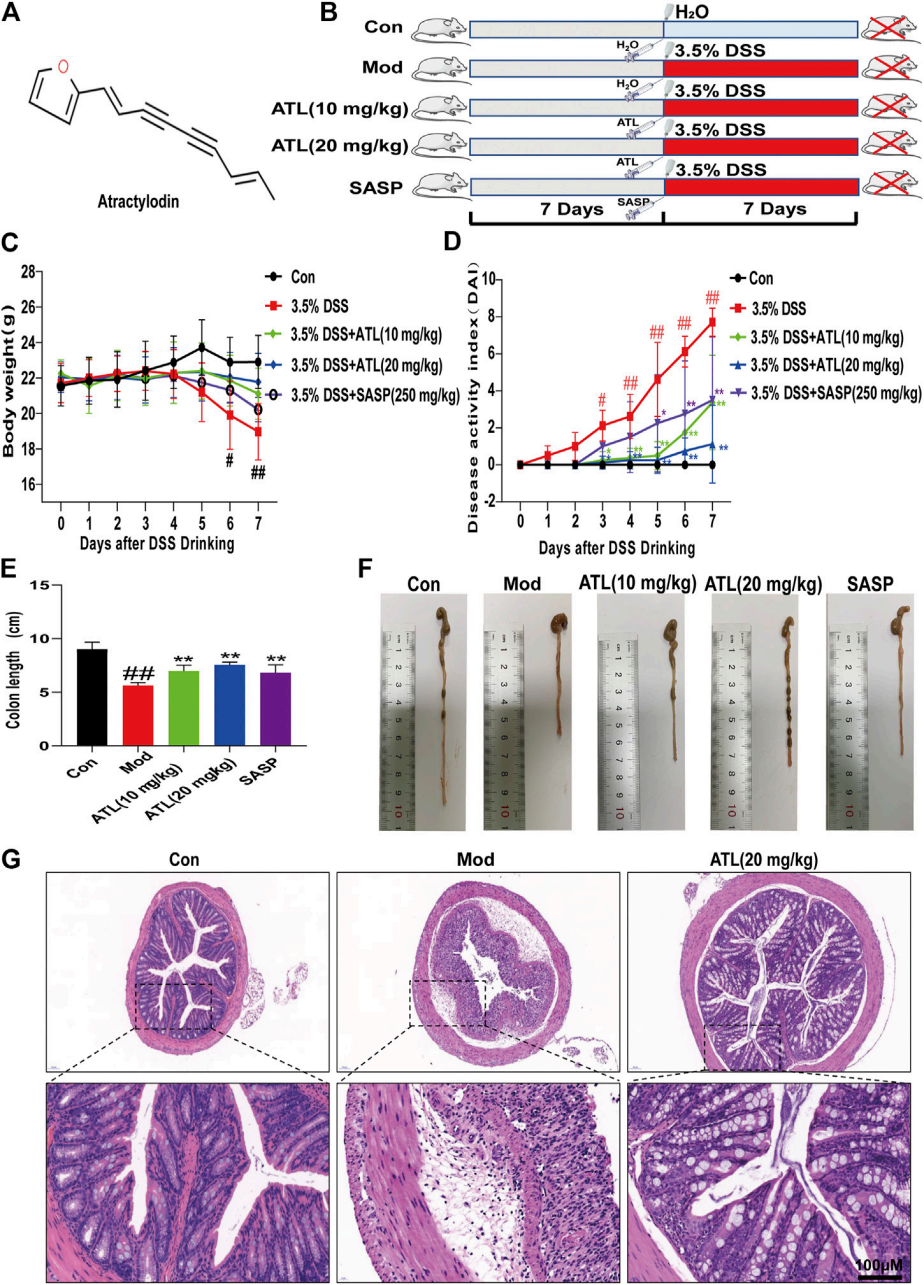
Atractylodin (ATL) attenuates symptoms of dextran sulfate sodium-induced colitis in mice **(A)** Structure of ATL **(B)** Schematic diagram of animal experimental design. Control (CON), model (MOD), atractylodin (ATL), sulfasalazine (SASP) **(C)** Daily body weights of mice **(D)** Calculated disease activity index scores **(E,F)** Images of the intestine and statistics of colon length in each group **(G)** Representative HE-stained images of colon sections (× 400 magnification). Data are expressed as the mean ± SEM, *n* = 8. Data were analyzed using one-way ANOVA. #*p* < 0.05, ##*p* < 0.01 compared to the control group. *p < 0.05, **p < 0.01 compared to the model group.

The weight of mice that consumed DSS-infused water decreased significantly from the third day compared to those in the normal group. The weight loss of the ATL (10 or 20 mg/kg) intervention groups and SASP group was lower than that in the model group (Figure 1C). Compared to that of the normal group, the disease activity index (DAI) score of the model group was significantly different from the third day (*p* < 0.05) and significantly different from the fourth day (*p* < 0.01). Compared to that of the model group, the DAI scores of ATL (10 or 20 mg/kg) and SASP groups increased gradually, and there was a significant difference between the ATL (10 or 20 mg/kg) and model groups from the third day (*p* < 0.05). A significant difference was found between the SASP and model groups on the fifth day (*p* < 0.05) (Figure 1D). After DSS therapy, the colon lengths of mice were found to be significantly shortened (*p* < 0.01). The mice in the ATL (10 or 20 mg/kg) and SASP groups did not exhibit colon shortening. Compared to the mice in the model group, those in the ATL (10 or 20 mg/kg) and SASP groups exhibited significant changes (*p* < 0.01) (Figures 1E,F). After treatment with DSS, the colonic glands of mice were obviously disordered, the goblet cells were obviously absent, and considerable inflammatory infiltration was observed compared to mice in the normal group. After the administration of ATL (20 mg/kg), the arrangement of glands was more orderly than that in the model group; the morphology of goblet cells recovered and inflammatory infiltration was significantly reduced (Figure 1G).

### Atractylodin Can Improve the Intestinal Barrier of Mice With Ulcerative Colitis

**FIGURE 2:**
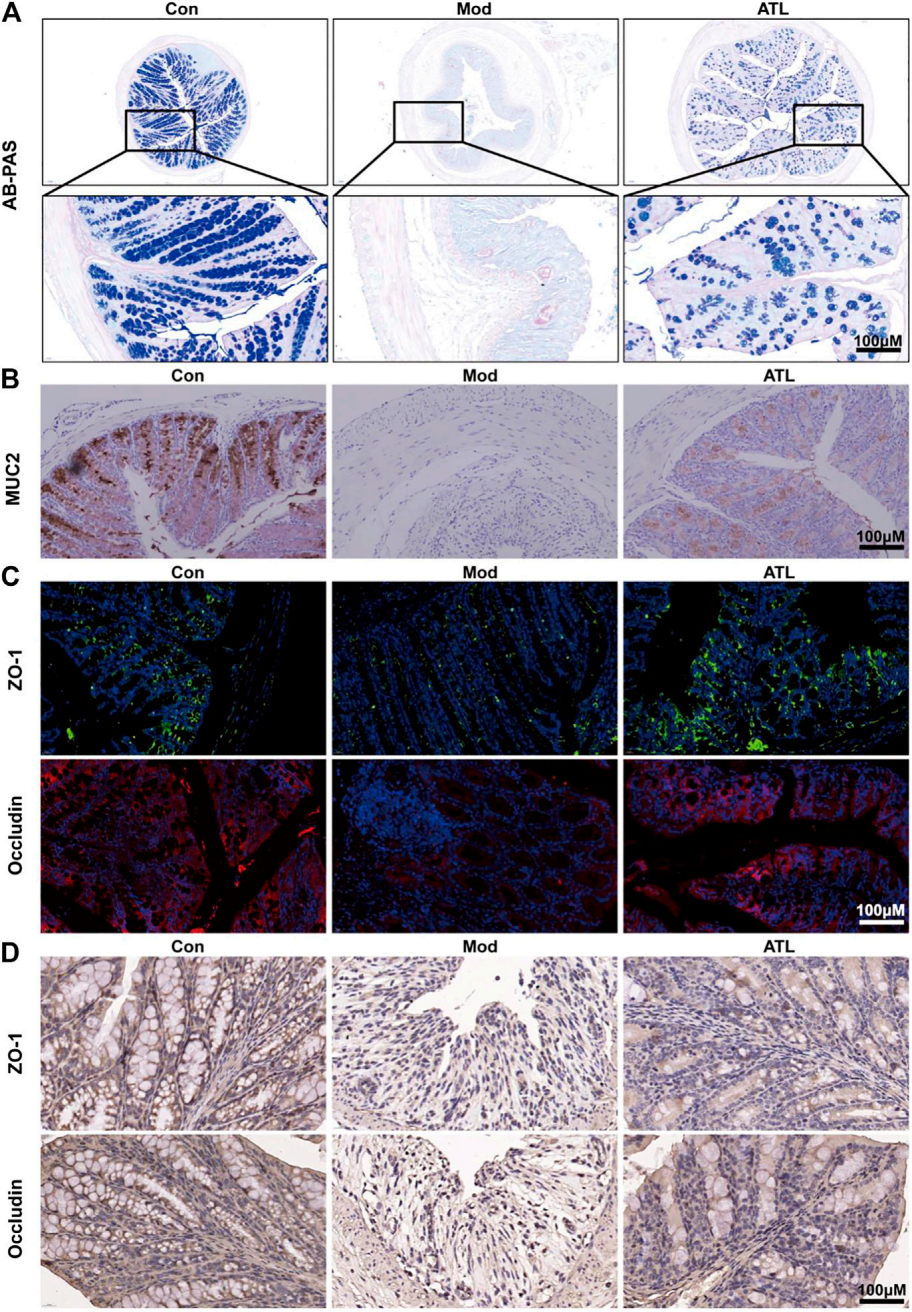
ATL attenuates intestinal barrier of DSS-induced colitis in mice **(A)** Representative Alcian Blue-Periodic Acid Schiff (AB-PAS) images of colon sections **(B)** Representative immunostaining images of colon sections stained for Mucin-2 (MUC2) **(C)** Protein expression of zona occludens-1 (ZO-1) and occludin from colon tissue **(D)** Representative immunostaining images of colon sections stained for ZO-1 and occludin.

The number of goblet cells in the colon of mice that consumed DSS decreased significantly compared to that in the normal group. After treatment with ATL (20 mg/kg), the number of colonic goblet cells increased significantly (Figure 2A). The expression of MUC2 in the colons of DSS-induced mice was found to be significantly decreased. After atractylodin (20 mg/kg) treatment, the expression of MUC2 in the colon increased significantly (Figure 2B). The expression of ZO-1 and occludin in the colons of DSS-induced mice decreased significantly. After treatment with ATL (20 mg/kg), the expression of tight junction proteins ZO-1 and occludin increased significantly (Figures 2C,D).

### Atractylodin can Improve the Intestinal Inflammation of Mice With UC

**FIGURE 3:**
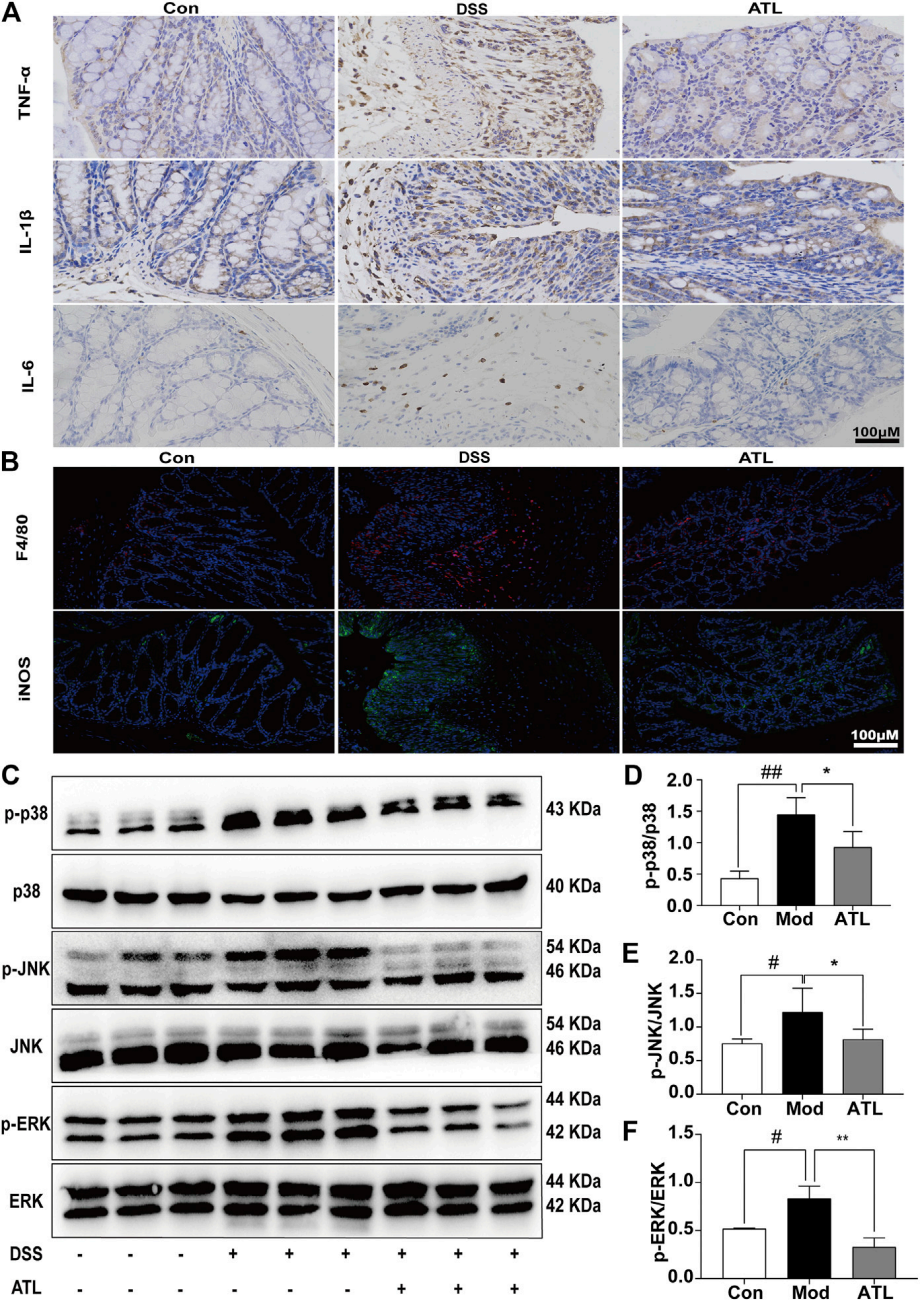
ATL attenuates intestinal inflammation of DSS-induced colitis in mice **(A)** Immunohistochemistry of inflammatory cytokines (TNF-α, IL-6, and IL-1β) **(B)** Immunofluorescent staining of macrophages (F4/80, iNOS) **(C)** Representative western blots of p-p38, p38, *p*-JNK, JNK, *p*-ERK, and ERK in colon tissue **(D–F)** Relative density of each signaling band was calculated. Data are expressed as the mean ± SEM, *n* = 3. Data were analyzed using one-way ANOVA. #*p* < 0.05, ##*p* < 0.01 compared to the control group. *p < 0.05, **p < 0.01 compared to the model group.

The levels of pro-inflammatory cytokines, TNF-α, IL-1β, and IL-6, were significantly increased in mice that consumed DSS-infused water. After treatment with ATL (20 mg/kg), the levels of the pro-inflammatory cytokines were found to be significantly decreased (Figure 3A). Moreover, in DSS-induced mice, F480 and iNOS were determined to be significantly increased, whereas these levels decreased significantly after atractylodin treatment (Figure 3B). In DSS-induced mice, expression of the MAPK pathway proteins, such as phosphorylated p38, phosphorylated JNK, and phosphorylated ERK, was significantly upregulated in the colon tissue (Figure 3C). After treatment with ATL (20 mg/kg), phosphorylated p38 (Figure 3D), phosphorylated JNK (Figure 3E), and phosphorylated ERK levels (Figure 3F) in the colon tissue were significantly downregulated.

### Atractylodin can Inhibit the MAPK Pathway and the Expression of Inflammatory Factors in Macrophages

**FIGURE 4:**
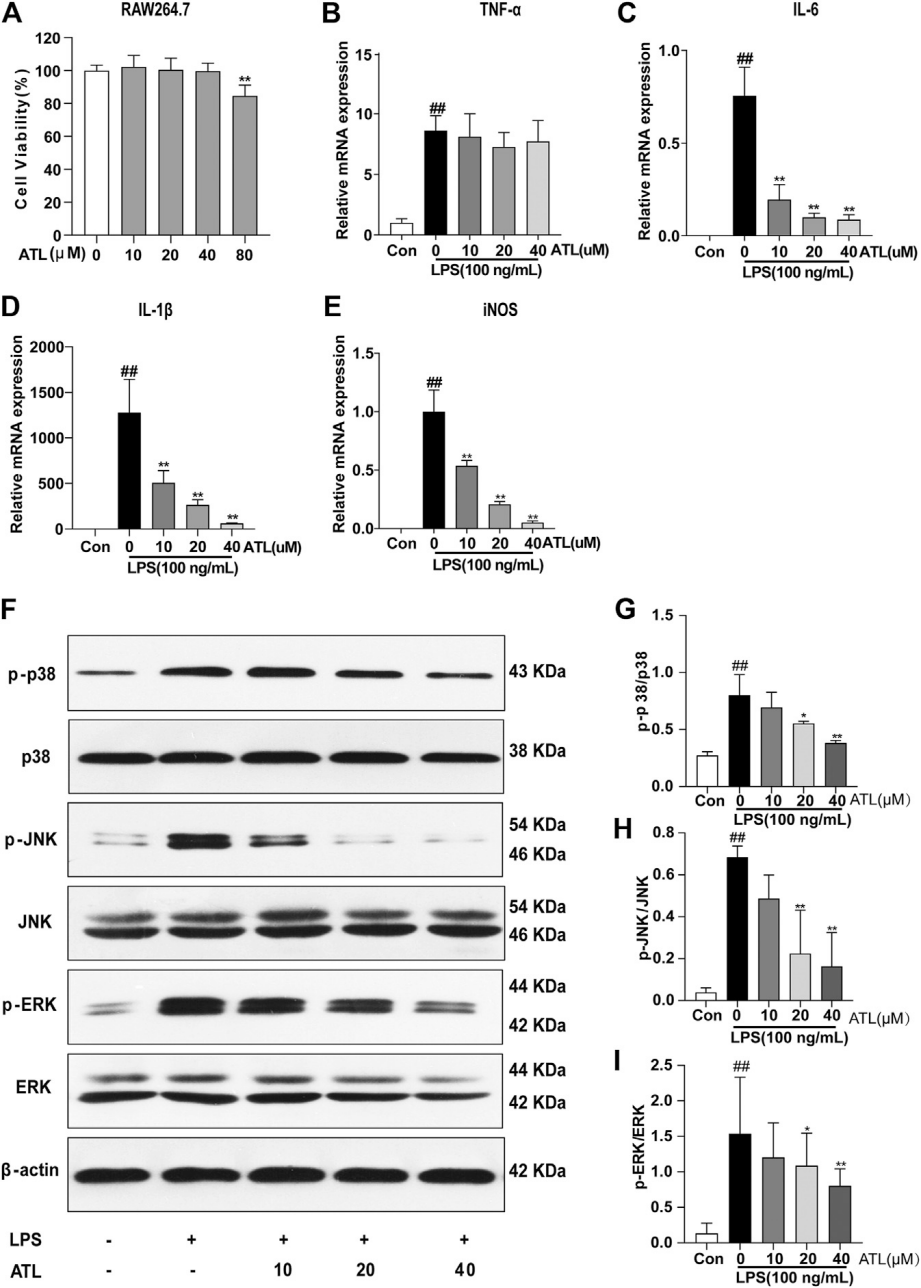
ATL can inhibit the MAPK pathway and expression of inflammatory factors in macrophages **(A)** Cell viability of RAW264.7 cells treated without or with different concentrations of ATL (B–E) mRNA levels of tumor necrosis factor alpha (TNF-α) **(B)** interleukin 6 (IL-6) **(C)**, interleukin-1*β* (IL-1*β*) **(D)** and inducible nitric oxide synthase (iNOS) **(E)** in RAW264.7 cells subjected to LPS (lipopolysaccharide) (100 ng/ml) stimulation and ATL treatment **(F)** Representative western blots of p-p38, p38, *p*-JNK, JNK, *p*-ERK, and ERK in RAW264.7 cells **(G–I)** Relative density of each signaling band was calculated. Data were obtained from three independent experiments and are presented as the mean ± SEM. Data were analyzed using one-way ANOVA, *n* = 3. #*p* < 0.05, ##*p* < 0.01 compared to the control group. *p < 0.05, **p < 0.01 compared to the LPS (100 ng/ml) + 0 μM ATL group.

Cell viability assays showed that atractylodin was not cytotoxic to RAW264.7 cells at a concentration of 0–40 μM (Figure 4A). The mRNA levels of TNF-α, IL-1β, IL-6, and iNOS in RAW264.7 cells stimulated by LPS (100 ng/ml) for 24 h were significantly increased. The expression of IL-1β, IL-6, and iNOS decreased by varying degrees after 24 h of atractylodin intervention (Figures 4B,C–E). The MAPK pathway proteins, including phosphorylated p38, phosphorylated JNK, and phosphorylated ERK, in the LPS-induced macrophages were upregulated (Figure 4F). After treatment with different concentrations of atractylodin, phosphorylated p38 (Figure 4G), phosphorylated JNK (Figure 4H), and phosphorylated ERK (Figure 4I) in the macrophages were downregulated.

### Atractylodin Can Target GAPDH and Inhibit the Inflammatory Process

**FIGURE 5:**
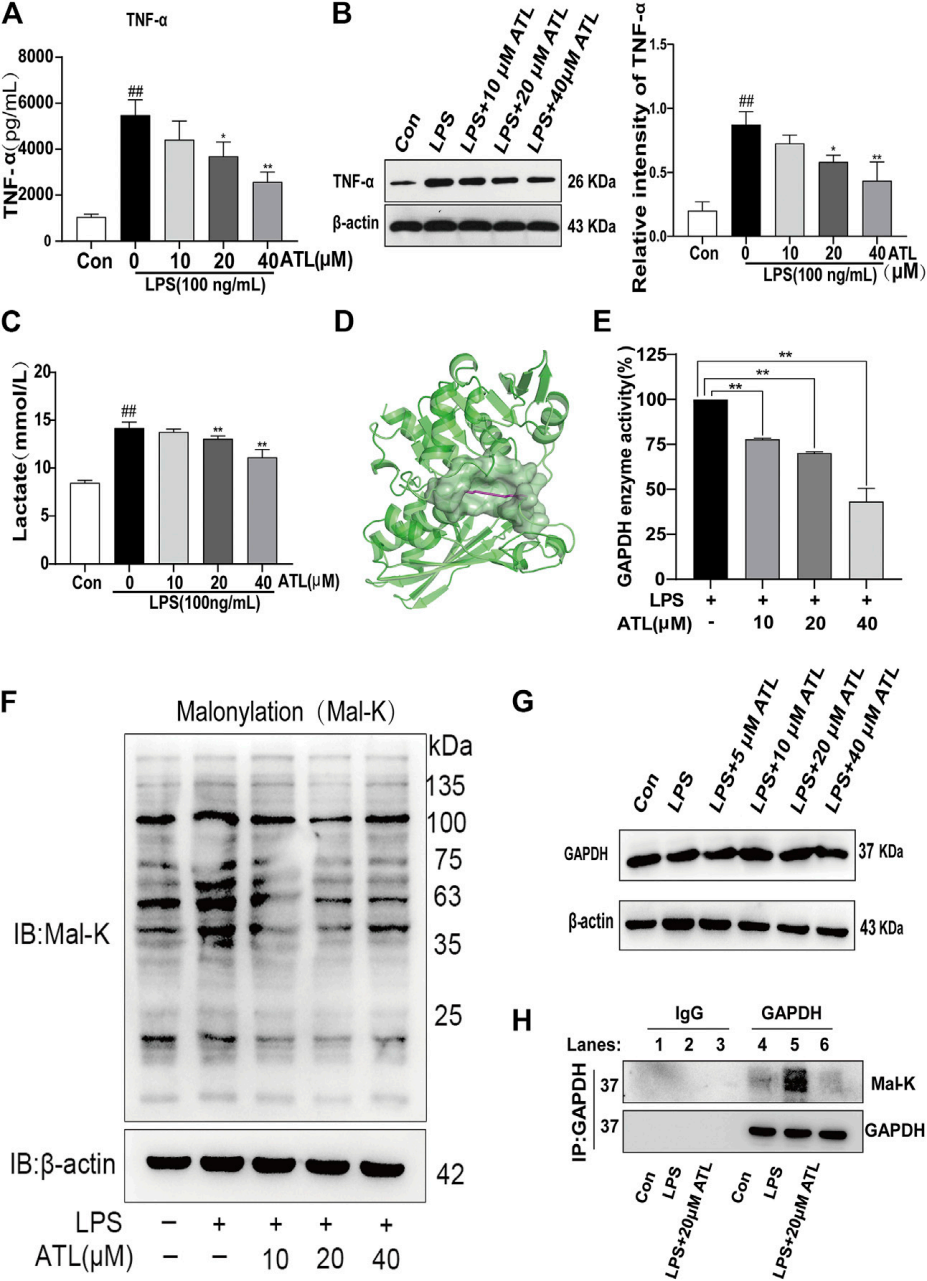
ATL targets GAPDH to inhibit the inflammatory process **(A)** TNF-α levels in the supernatant of RAW264.7 cells were measured using ELISA (enzyme linked immunosorbent assay) **(B)** TNF-α levels in the lysate of RAW264.7 cells were measured using western blotting **(C)** Lactate levels in the supernatant of RAW264.7 cells were measured using a lactate assay kit **(D)** Molecular docking of ATL with GAPDH **(E)** GAPDH enzyme activity in the lysate of RAW264.7 cells was measured using a GAPDH enzyme activity kit **(F)** Western blot analysis to determine lysine malonylation (mal-K) in the lysates of RAW264.7 treated with LPS (100 ng/ml) for 24 h **(G)** GAPDH expression in LPS-treated RAW264.7 cells was analyzed using western blotting **(H)** Immunoprecipitated GAPDH from the control, LPS-treated (100 ng/ml), and LPS (100 ng/ml) + ATL (20 μM)-treated RAW264.7 cells and samples probed with an anti-mal-K antibody (lower panel). GAPDH expression in the immunoprecipitated (upper panel) samples was also examined. Data were obtained from three independent experiments and are presented as the mean ± SEM. Data were analyzed using one-way ANOVA, *n* = 3. #*p* < 0.05, ##*p* < 0.01 compared to the control group. *p < 0.05, **p < 0.01 compared to the LPS (100 ng/ml) + 0 μM ATL group.

TNF-α was found to be highly expressed in the supernatant and lysate of LPS-treated macrophages; however, its levels were decreased by varying degrees after treatment with different concentrations of atractylodin. The expression of TNF-α protein significantly decreased after treatment with 20 μM (*p* < 0.05) atractylodin and significantly decreased at 40 μM (*p* < 0.01) (Figures 5A,B) of atractylodin. Lactate levels in the supernatant of the LPS-treated macrophages were found to be significantly increased, whereas treatment with different concentrations of atractylodin led to a decrease. Lactate levels were significantly decreased after treatment with 20 μM (*p* < 0.05) of atractylodin and extremely significantly decreased upon treatment with 40 μM (*p* < 0.01) of the compound (Figure 5C). Molecular docking experiments with atractylodin and GAPDH revealed binding free energy of 5.42 kcal/mol (Figure 5D) and suggested that GAPDH was likely an anti-inflammatory target of atractylodin. We investigated the effects of atractylodin on GAPDH activity and found that its activity decreased gradually with an increase in atractylodin concentration (Figure 5E). However, different concentrations of atractylodin did not affect the expression of the GAPDH protein (Figure 5G). In LPS-treated macrophages, malonylation of the total protein was significantly enhanced, and we found that atractylodin could inhibit this process (Figure 5F). Results from the immunoprecipitation assay revealed that atractylodin could significantly inhibit malonylation of the GAPDH protein (Figure 5H).

### Atractylodin Can Improve the Intestinal Microbiota of Mice With DSS-Induced Ulcerative Colitis

**FIGURE 6:**
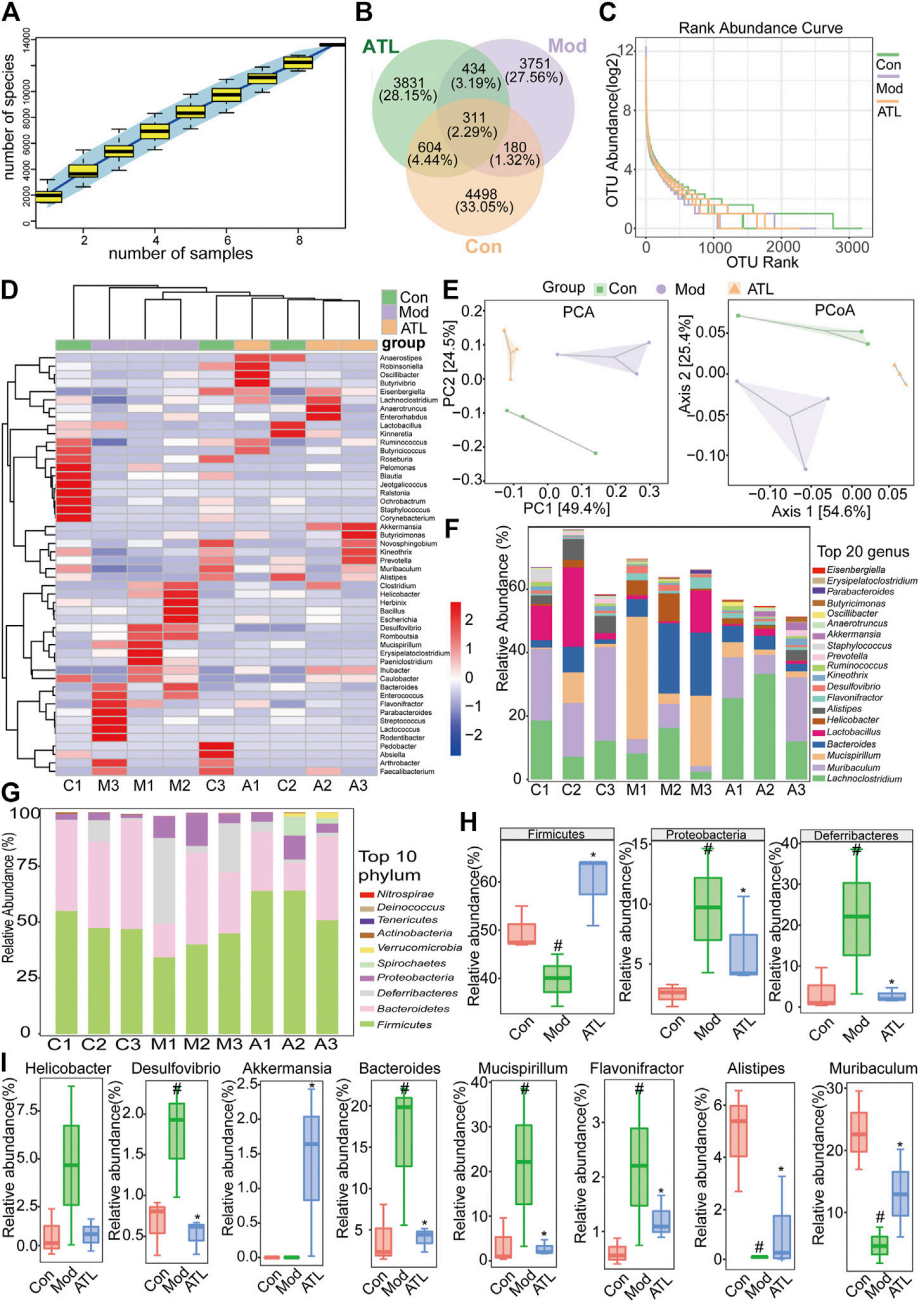
ATL can improve the intestinal microbiota of DSS-induced colitis in mice **(A)** Sample number and species richness were estimated from a species accumulation boxplot **(B)** Venn diagram of species **(C)** Abundance grade curve of species level **(D)** Heatmap demonstrating the relative abundance of intestinal bacteria at the genus level **(E)** PCA (principal component analysis) and PCoA (principal coordinates analysis) demonstrated distinct structural changes in the overall bacterial community for each group **(F)** Microbial community bar plot at the genus level **(G)** Microbial community bar plot at the phylum level **(H)** Relative abundance of different microbial flora at the phylum level and the genus level **(I)** Data were obtained from three independent experiments and are presented as the mean ± SEM. Data were analyzed using one-way ANOVA, *n* = 3. #*p* < 0.05 compared to the control group. *p < 0.05 compared to the model group.

The cumulative species curve revealed that nearly 5,000 species of intestinal microorganisms were present in the three samples, which could essentially cover the common species (Figure 6A). The number of common intestinal bacteria in the normal and atractylodin (20 mg/kg) group was significantly higher than that in the normal and model group (Figure 6B). The grade curve at the species level showed that the abundance of the intestinal microflora was the most in the normal group and the least in the model group. The abundance of intestinal microflora in the atractylodin (20 mg/kg) group was midway between the normal and model groups (Figure 6C). Cluster analysis revealed that the microflora composition of the normal and model groups was significantly separated, whereas that of the atractylodin (20 mg/kg)-treated groups was close to the composition of the normal group (Figure 6D). Principal component analysis and principal coordinate analysis showed that the distribution of intestinal flora in the normal and model groups was well separated, whereas the atractylodin (20 mg/kg) and normal groups were closely clustered (Figure 6E). The distribution maps of the top 20 genera and top 20 phyla with the highest abundance among different groups of mice in this study are shown in Figures 6F,G, respectively. The abundance of *Firmicutes* decreased significantly after DSS treatment but increased significantly in the atractylodin (20 mg/kg) group. After DSS treatment, the abundance of Proteobacteria and Deferribacteres increased significantly, whereas treatment with atractylodin (20 mg/kg) resulted in a significant decrease in their abundance (Figure 6H). After DSS modeling, the abundance of pathogenic bacteria, including *Helicobacter, Desulfovibrio, Bacteroides, Flavonifractor, and Mucispirillum* increased significantly, while that of beneficial bacteria, including *Alistipes and Muribaculum,* decreased significantly. Atractylodin (20 mg/kg) treatment not only decreased the abundance of these pathogenic bacteria but also increased the abundance of beneficial bacteria including *Akkermansia* (Figure 6I).

## Discussion

Ulcerative colitis is a debilitating inflammatory disease of the intestine, which may increase the incidence of colon cancer, eventually leading to disturbances in the physical and mental health of individuals (Pandurangan and Esa, 2014). Corticosteroids, aminosalicylates, biological agents, and immunosuppressants can alleviate UC-related symptoms and inflammation. However, these drugs are associated with adverse effects (Zhu et al., 2021). Therefore, safe therapeutic alternatives in the management of UC are much needed. One of the approaches in drug development is to isolate natural products from traditional Chinese herbal medicine and plants and evaluate them for the treatment of UC.

Our results indicated that atractylodin could reduce the weight loss of mice with UC, control diarrhea and hematochezia, alleviate trauma to the colon, reduce the loss of colonic goblet cells, and increase the expression of MUC2, ZO-1, and occludin.

Macrophages play an important role in regulating the homeostasis of the intestinal immune microenvironment. During inflammation, intestinal macrophages are activated to pro-inflammatory phenotype M1 type, which can upregulate TNF-α, IL-1β, IL-6, and other inflammatory cytokines, increase the levels of reactive oxygen species, and aggravate inflammation (Zhao et al., 2020). F4/80 and iNOS are common macrophage markers (Wu et al., 2020). MAPK pathway is key for macrophage activation and plays an important role in affecting the progress of UC (Jing et al., 2019). Our findings indicated that atractylodin could inhibit the activation of macrophages in colonic tissue of mice with UC; reduce the production of TNF-α, IL-1β, and IL-6 in colonic tissue of mice with UC; and inhibit the activation of the inflammatory MAPK pathway both *in vivo* and *in vitro* in RAW264.7 cells.

Under inflammatory conditions, the energy metabolism of macrophages transforms from oxidative phosphorylation to glycolysis, thus rapidly producing a large amount of energy, which is similar to the Warburg effect observed in tumors (Yang et al., 2014). Glycolysis metabolism promotes the survival, differentiation, and effector function of activated macrophages (Van et al., 2015). Lactate is the end product and an important marker of glycolysis (Guo et al., 2015). GAPDH is a rate-limiting enzyme in activated macrophages, which can regulate the rate of erobic glycolysis (Shestov et al., 2014). A decrease in GAPDH activity can inhibit glycolysis and the inflammatory response of macrophages (Liao et al., 2019). Post-translational modification, which has a great impact on the function of proteins in health and disease, is key to expand the functional diversity of proteins (Karve and Cheema, 2011). Malonylation is a recently discovered, evolutionarily conserved modification (Peng et al., 2011). Malonylation of several proteins occurs in LPS-stimulated macrophages. In resting macrophages, GAPDH binds to the mRNA encoding TNF-α, thereby inhibiting the translation of TNF-α mRNA. Upon stimulation with LPS, GAPDH protein undergoes malonylation, which separates it from the mRNA encoding TNF-α and promoting the translation of TNF-α mRNA (Galván-Pea et al., 2019). The findings of our study showed that atractylodin could inhibit lactate production, GAPDH activity, and the malonylation of the GAPDH protein, which is a likely explanation of why this compound could not inhibit transcription but could inhibit the translation of TNF-α.

Intestinal flora plays an important role in the pathogenesis of UC and may determine the severity of intestinal inflammation (Scaldaferri et al., 2013). Normal intestinal microflora can produce metabolites in the gut that are not conducive to the colonization of intestinal pathogens. Antibiotics, dietary changes, and certain drugs and diseases can prevent the secretion of metabolites, resulting in the proliferation of pathogens, which consequently trigger inflammatory reactions (Stecher, 2015). The physical barrier comprises two layers of colonic mucus, which protects the host against several intestinal bacteria. The mucous layer of the large intestine is thick and comprises the inner mucous layer and the outer loose mucous layer. Studies show that the inner mucous layer and its barrier function are important factors limiting contact between bacteria and the gut epithelium, and their disorders may cause inflammation (Okumura and Takeda, 2017). Moreover, intestinal microbiota can alter the properties of the colonic mucous layer. Rupture of the intestinal mucus layer leads to the invasion of intestinal bacteria, which eventually leads to inflammation and infection (Schroeder, 2019). The gut contains immune cells that can recognize and eliminate antigens. Intestinal epithelial cells receive signals from intestinal flora via pathogen recognition receptors and convert these signals into mucosal immune signals (Martini et al., 2017). An imbalance in the intestinal flora results in a considerable decrease in the function of the mucosal barrier, rendering the intestinal submucosa susceptible to invasion by pathogenic bacteria and activation of the microbial antigens. Collectively, this leads to immune imbalance, inflammatory-cell activation, and an inflammatory reaction in the host. Therefore, pharmacological therapies aimed at maintaining the intestinal microbiota are gaining increased interest (Gophna et al., 2006).

An increase in the relative abundance of Firmicutes using fecal microbial transplantation resulted in alleviation of the symptoms of UC in patients, as increased microbial groups of Firmicutes are mostly related to healthy intestines (Schierová et al., 2020). Proteobacteria is one of the larger bacterial communities, which includes several pathogenic bacteria, such as *Escherichia coli*, *Salmonella*, *Vibrio cholerae*, and *Helicobacter pylori* among others. The proportion of Proteobacteria in patients with ulcerative colitis is known to increase significantly (Xu et al., 2021). Adhesively invasive *E. coli* increased the abundance of Proteobacteria and Deferrobacter in a porcine model of experimental colitis and these findings were similar to that observed in patients with IBD (Munyaka et al., 2016). The results of our study indicated that atractylodin could significantly increase the abundance of Firmicutes and significantly reduce that of *Proteus* and *Deferrobacteria*. At the genus level, atractylodin could significantly inhibit the abundance of pathogenic bacteria, such as *Helicobacter*, *Vibrio desulfuricus*, *Bacteroides*, *Flavobacterium*, *and Mucispirillum*, and increase that of beneficial bacteria including *Alistipes*, *Muribaculum*, and *Akkermansia*.

**FIGURE 7:**
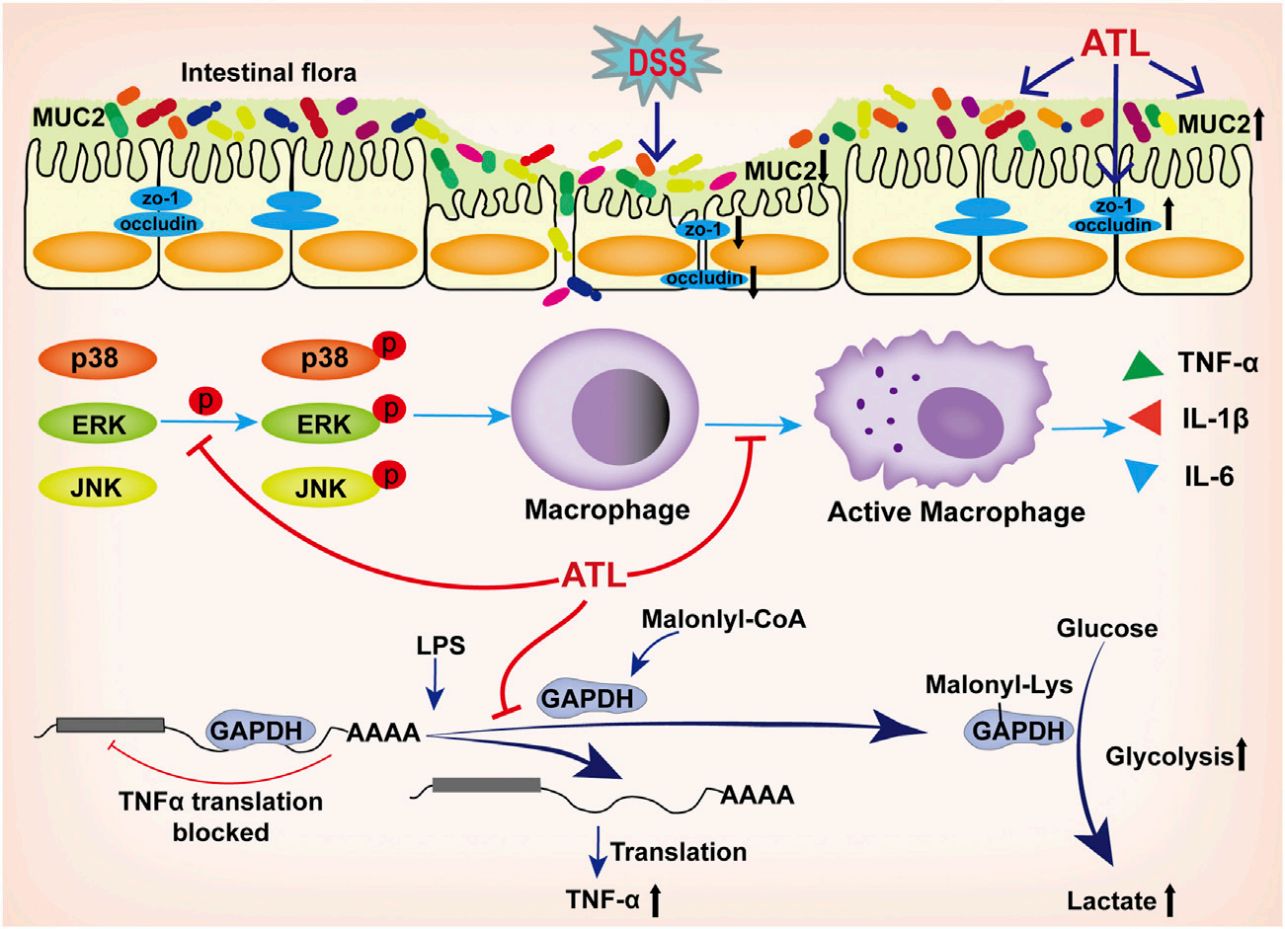
Atractylodin attenuates dextran sulfate sodium-induced colitis by alleviating gut microbiota dysbiosis and inhibiting inflammatory response through the MAPK pathway. The mechanism could likely be attributed to the effect of atractylodin in inhibiting the MAPK pathway; inhibiting the activation of macrophages in colon tissue; inhibiting the decrease in tight junction proteins, ZO-1, occludin, and MUC2 to protect the intestinal barrier; decreasing the abundance of harmful bacteria and increasing that of beneficial bacteria, thereby improving the overall intestinal microflora. *In vitro* experiments revealed that atractylodin could inhibit the MAPK pathway and macrophage activation. Atractylodin could not inhibit the transcription of TNF-α but could inhibit its translation, which might be related to its effect of inhibiting the malonylation of the GAPDH protein.

To summarize, atractylodin could significantly improve the progression of DSS-induced ulcerative colitis. The mechanism could likely be attributed to the effect of atractylodin in inhibiting the MAPK pathway; inhibiting the activation of macrophages in colon tissue; inhibiting the decrease in tight junction proteins, ZO-1, occludin, and MUC2 to protect the intestinal barrier; and decreasing the abundance of harmful bacteria and increasing that of beneficial bacteria, thereby improving the overall intestinal microflora. *In vitro* experiments revealed that atractylodin could inhibit the MAPK pathway and macrophage activation. Moreover, we found that atractylodin could not inhibit the transcription of TNF-α but could inhibit its translation, which might be related to its effect of inhibiting the malonylation of the GAPDH protein (Figure 7). Overall, the findings of our study indicated that atractylodin may be a potentially effective compound in the management of ulcerative colitis and, therefore, warrants further exploration.

## Data Availability

The data presented in the study are deposited in the Sequence Read Archive (SRA) 417 database of NCBI repository, accession number PRJNA715347.
