# Supplementary material for: Atractylodin Attenuates Dextran Sulfate Sodium-Induced Colitis by Alleviating Gut Microbiota Dysbiosis and Inhibiting Inflammatory Response Through the MAPK Pathway
Source: Front Pharmacol. 2021 Jul 15;12:665376. doi: 10.3389/fphar.2021.665376 (PMC8320761; doi:10.3389/fphar.2021.665376)
Supplement: Supplementary file 1 [file Table1.DOCX]

| IL-1β（F） | mouse | CTTCAGGCAGGCAGTATCACTC |
| --- | --- | --- |
| IL-1β（R） | mouse | TGCAGTTGTCTAATGGGAACGT |
| TNF-α（F） | mouse | CCT CTA GCCCAC GTC GTA GC |
| TNF-α（R） | mouse | AGCAATGACTCCAAAGTAGAC C |
| IL-6 (F) | mouse | ACAACCACGGCCTTCCCTAC |
| IL-6 (R) | mouse | TCTCATTTCCACGATTTCCCAG |
| iNOS (F) | mouse | TGCCACGGACGAGACGGATAG |
| iNOS (R) | mouse | CTCTTCAAGCACCTCCAGGAAC |
| Actin(F) | mouse | TGCTGTCCCTGTATGCCTCT |
| Actin(R) | mouse | TTTGATGTCACGCACGATTT |

S1 Primers used in RT-PCR
